# A longitudinal analysis of the vaginal microbiota and vaginal immune mediators in women from sub-Saharan Africa

**DOI:** 10.1038/s41598-017-12198-6

**Published:** 2017-09-20

**Authors:** Vicky Jespers, Jordan Kyongo, Sarah Joseph, Liselotte Hardy, Piet Cools, Tania Crucitti, Mary Mwaura, Gilles Ndayisaba, Sinead Delany-Moretlwe, Jozefien Buyze, Guido Vanham, Janneke H. H. M. van de Wijgert

**Affiliations:** 1 HIV and Sexual Health Group, Department of Public Health, Institute of Tropical Medicine, Antwerp, Belgium; 2 Virology Unit, Department of Biomedical Sciences, Institute of Tropical Medicine, Antwerp, Belgium; 3 MRC Clinical Trials Unit at University College London, London, UK; 4 Laboratory Bacteriology Research, Ghent University, Ghent, Belgium; 5 HIV/STI Reference Laboratory, Department of Clinical Sciences, Institute of Tropical Medicine, Antwerp, Belgium; 6 International Centre for Reproductive Health in Mombasa, Mombasa, Kenya; 7 Rinda Ubuzima, Kigali, Rwanda; 8 Wits Reproductive Health and HIV Institute, Johannesburg, South Africa; 9 Clinical Trials Unit, Department of Clinical Sciences, Institute of Tropical Medicine, Antwerp, Belgium; 10 Department of Clinical Infection, Microbiology and Immunology, Institute of Infection and Global Health, University of Liverpool, Liverpool, UK

## Abstract

In cross-sectional studies increased vaginal bacterial diversity has been associated with vaginal inflammation which can be detrimental for health. We describe longitudinal changes at 5 visits over 8 weeks in vaginal microbiota and immune mediators in African women. Women (N = 40) with a normal Nugent score at all visits had a stable lactobacilli dominated microbiota with prevailing *Lactobacillus iners*. Presence of prostate-specific antigen (proxy for recent sex) and being amenorrhoeic (due to progestin-injectable use), but not recent vaginal cleansing, were significantly associated with microbiota diversity and inflammation (controlled for menstrual cycle and other confounders). Women (N = 40) with incident bacterial vaginosis (Nugent 7–10) had significantly lower concentrations of lactobacilli and higher concentrations of *Gardnerella vaginalis*, *Atopobium vaginae*, and *Prevotella bivia*, at the incident visit and when concentrations of proinflammatory cytokines (IL-1β, IL-12p70) were increased and IP-10 and elafin were decreased. A higher ‘composite-qPCR vaginal-health-score’ was directly associated with decreased concentrations of proinflammatory cytokines (IL-1α, IL-8, IL-12(p70)) and increased IP-10. This longitudinal study confirms the inflammatory nature of vaginal dysbiosis and its association with recent vaginal sex and progestin-injectable use. A potential role for proinflammatory mediators and IP-10 in combination with the vaginal-health-score as predictive biomarkers for vaginal dysbiosis merits further investigation.

## Introduction

The vaginal mucosal surface is colonised by a variety of bacterial species and the composition, which has implications for reproductive health, is influenced by both endogenous and exogenous factors (reviewed in^1^). Using culture-dependent and molecular amplification techniques (such as quantitative polymerase chain reaction (qPCR) and next generation sequencing), a ‘normal’ vaginal microbiota (VMB) has been defined as one dominated by lactic acid-producing *Lactobacillus* species. The clinical condition bacterial vaginosis (BV) is associated with increased diversity and quantity of bacteria and a concomitant decrease in lactobacilli^2^. Molecular studies have shown some lactobacilli (notably *L. crispatus*) are more associated with health than others (*L. iners*) because they are associated with a lower risk of developing vaginal dysbiosis^3^ or acquiring sexually transmitted infections (STIs)^4^.

BV is the most common vaginal dysbiosis^1^ and has been associated with adverse clinical outcomes including pre-term birth and miscarriage^5^, pelvic inflammatory disease^6^, and the acquisition and transmission of STIs including HIV^7–10^. It has been suggested that such adverse outcomes are directly associated with inflammatory or immune activation cascades triggered by vaginal dysbiosis^11^. For example, inflammation triggered by vaginal dysbiosis probably attracts CD4 + cells to the cervicovaginal mucosa, thereby increasing the availability of target cells for HIV at the site of viral entry into the body^12^.

Sub-Saharan Africa has the highest prevalence of BV^13^ but diagnosis is often missed because symptoms are frequently absent or nonspecific, and the microscopic methods necessary for diagnosis by Amsel criteria or Nugent^14^ scoring are typically not available. Cross-sectional studies using molecular techniques have confirmed the high prevalence of vaginal dysbiosis in sub-Saharan Africa and have also shown that *L. iners* dominated VMB are more frequent than those dominated by *L. crispatus* ^15,16^. Sub-Saharan African women may therefore be less protected from vaginal dysbiosis, even when they have a lactobacilli-dominated VMB.

Information on fluctuations in the VMB over time is limited, but studies^17,18^ have shown the variation within an individual over time is more pronounced than the variation between individuals^19^, and that such changes are influenced by the menstrual cycle and can occur rapidly^17,20^.

We conducted a longitudinal cohort study (the Vaginal Biomarkers Study) in 430 women at three sites in Kenya, Rwanda and South Africa^21^. The study visits were tightly scheduled to control for the menstrual cycle. We selected 40 women with consistently normal VMB (defined as a Nugent score of 0–3) at five study visits over eight weeks and 40 women who developed BV (Nugent score of 7–10) during the same eight-week period. The primary objective of this sub-study was to describe the vaginal bacterial species and concentrations of vaginal immune mediators in these cohorts over time. Secondary objectives included the determination of host correlates of vaginal bacteria and immune mediators, any associations between them and those which occurred around the time of incident BV diagnosis. This is the first longitudinal study to describe the composition of the VMB and vaginal immune mediators in quantitative terms over time as well as any associations between them whilst controlling for menstrual cycle and other factors known to be associated with changes in the vaginal micro-environment.

## Results

The cross-sectional characteristics of all 430 women enrolled in the Vaginal Biomarkers Study, including the composition of the VMB, have been previously described^15,16,21,22^. In this sub-study, the median age of the 40 women with a consistently normal VMB (reference group) and the 40 women who developed incident BV (incident BV group) was 23 and 24 years respectively (ranges 16–34 years and 16–33 years). The median age at first vaginal intercourse was 17 years for both groups. Half of the women with a consistently normal VMB (53%) and 43% of the women who developed BV had had two or three lifetime sex partners with most (78% and 88%, respectively) having had one sex partner in the last three months. Over fifty percent of the women in both groups had delivered a child at least once. Most women in both groups (80%) currently used contraception: 36% used progestin injections, 13% used combined hormonal pills, 5% were sterilised, and 26% used condoms only. All women in the reference group tested negative for pregnancy, HIV, syphilis, *Neisseria gonorrhoeae*, *Chlamydia trachomatis*, and *Trichomonas vaginalis*, by design. The baseline herpes simplex virus type 2 (HSV-2) prevalence was 30% in the women with a consistently normal VMB and 33% in the women who developed BV.

Table 1 describes participant characteristics by group over the five study visits for those parameters that were subsequently included in mixed effects regression models as potential confounders of the main associations of interest between VMB bacteria and vaginal immune mediators (see methods). The reference group included HIV-negative adult women (N = 16 Kenya, N = 16 South Africa), adolescents (N = 6 Kenya), and HIV-negative sex workers (N = 2 Rwanda). The incident BV group included HIV-negative adult women (N = 16 Kenya, N = 11 South Africa), adolescents (N = 5 Kenya, N = 2 South Africa), pregnant women (N = 1 Kenya, N = 3 South Africa); and HIV-negative sex workers (N = 2 Rwanda). The detection of prostate-specific antigen (PSA) in the vagina as a marker of vaginal sex in the last 24–48 hours^23,24^ and self-reported vaginal cleansing in the evening or morning just prior to the study visit were both common (25–57% and 28–53% at different visits, respectively). Clinician-observed abnormal vaginal discharge and cervical mucus were also common, but cervical epithelial findings visible by the naked eye (abrasion, laceration, ecchymosis, petechiae, erythema, or ulcer) were uncommon (occurring in 1–8 women at each visit), throughout the study (Table 1). At all visits, a substantial proportion of women (up to 33% of the women with a consistently normal VMB and up to 68% of the women who developed BV) had a vaginal pH above 4.5, which is considered outside the normal range and is one of the Amsel criteria for the diagnosis of BV^25^.

**Table 1:** The prevalence of participant characteristics at each study visit.

**Characteristics of women with a normal VMB throughout**	**Visit 1 (n = 40)**	**Visit 2 (n = 40)**	**Visit 3 (n = 40)**	**Visit 4 (n = 40)**	**Visit 5 (n = 40)**
Vaginal PSA present	11 (27.5)	11 (27.5)	10* (25.0)	11 (27.5)	16 (40.0)
Vaginal cleansing during bathing this morning or last night	16 (40.0)	12 (30.0)	11 (27.5)	11 (27.5)	11 (27.5)
Clinician-observed abnormal vaginal discharge	7 (17.5)	10 (25.0)	10 (25.0)	10 (25.0)	16 (40.0)
Clinician-observed cervical mucus present	13 (32.5)	11* (28.2)	15 (37.5)	17 (42.5)	16 (40)
Clinician-observed cervical epithelial abnormalities present†	6 (15.0)	4 (10.0)	4 (10.0)	4 (10.0)	8 (20.0)
Petechiae	5 (12.5)	3 (7.5)	2 (5.0)	3 (7.5)	3 (7.5)
Erythema	1 (2.5)	0	1 (2.5)	1 (2.5)	4 (10.0)
Ecchymosis	0	1 (2.5)	1 (2.5)	0	0
Ulcer	0	0	0	0	1 (2.5)
Vaginal pH < 4	8 (20.0)	9 (22.5)	13 (33.3)	11 (27.5)	9 (22.5)
4–4.5	19 (47.5)	20 (50.0)	17 (43.6)	16 (40.0)	22 (55.0)
4.6–5	10 (25.0)	9 (22.5)	6 (15.4)	11 (27.5)	7 (17.5)
>5	3 (7.5)	2 (5.0)	3 (7.5)	2 (5.0)	2 (5.0)
**Characteristics of women with incident BV**	**Visit 1 (n = 40)**	**Visit 2 (n = 40)**	**Visit 3 (n = 40)**	**Visit 4 (n = 40)**	**Visit 5 (n = 40)**
Vaginal PSA present	12 (30.8)	18 (48.7)	21 (56.8)	15 (40.5)	14 (43.8)
Vaginal cleansing during bathing this morning or last night	21 (52.5)	15 (38.5)	14 (35.0)	16 (41.0)	13 (33.3)
Clinician-observed abnormal vaginal discharge	5 (20.5)	6 (15.4)*	13 (32.5)	11 (28.2)*	11 (29.0)**
Clinician-observed cervical mucus present	14 (35.0)	10 (25.6)*	10 (25.0)	12 (30.8)*	14 (36.8)**
Clinician-observed cervical epithelial abnormalities present^ǁ^	5 (12.5)	3 (7.7)*	2 (5.0)	3 (7.7)*	3 (7.7)*
Petechiae	3 (7.5)	0	1 (2.5)	0	1 (2.6)
Ecchymosis	1 (2.5)	1 (2.6)	0	0	0
Erythema	0	1 (2.6)	0	1 (2.6)	2 (5.1)
Laceration	1 (2.5)	1 (2.6)	0	1 (2.6)	0
Ulcer	0	0	1 (2.5)	0	0
Abrasion	0	0	0	1 (2.6)	0
Vaginal pH < 4	4 (10.0)	4 (10.5)	3 (7.5)	1 (1.6)	3 (7.9)
4–4.5	22 (55.0)	16 (42.1)	10 (25.0)	16 (41.0)	10 (26.3)
4.6–5	12 (30.0)	8 (21.1)	13 (32.5)	16 (41.0)	16 (42.1)
>5	2 (5.0)	10 (26.3)	14 (35.0)	6 (15.4)	9 (23.7)

Abbreviations: PSA = prostate specific antigen; VMB = vaginal microbiota. Data are number of women with the characteristic (% of total number of women). Cervical mucus presence includes mild, moderate or abundant mucus. * One missing value. ** Two missing values. † 26 events in 13 participants. ^ǁ^16 events in ten participants.

### VMB bacteria and Candida over time (reference group)

**Figure 1:**
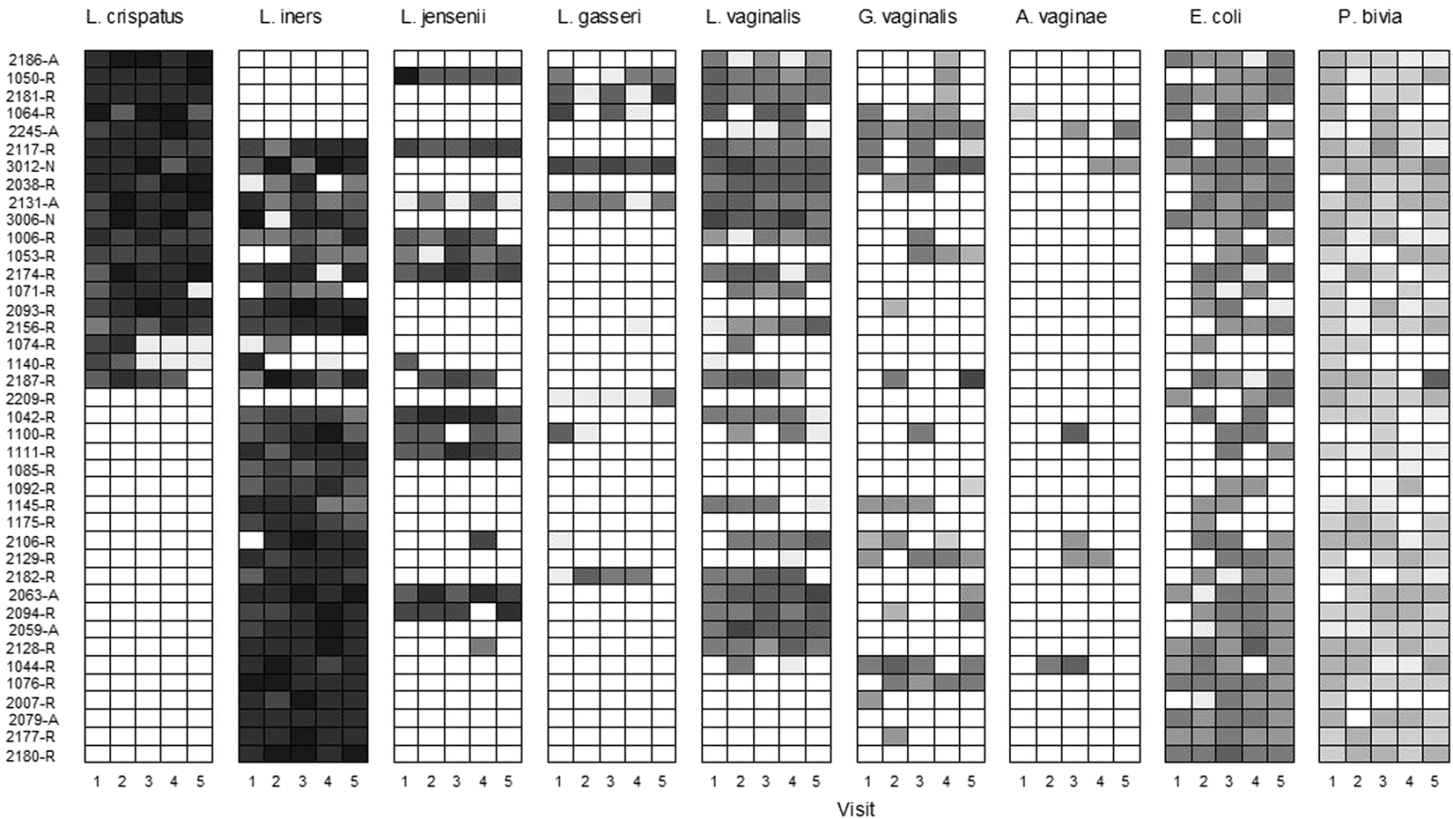
Presence/absence and concentration of vaginal microbiota bacteria over the eight week study period in women with a Nugent score of 0–3 throughout. Each box depicts one visit for a particular woman. The shading of the box indicates the concentration (in log_10_ geq/ml) of each taxon with darker colours depicting a higher concentration. If the taxon was absent, the box is white.

**Figure 2:**
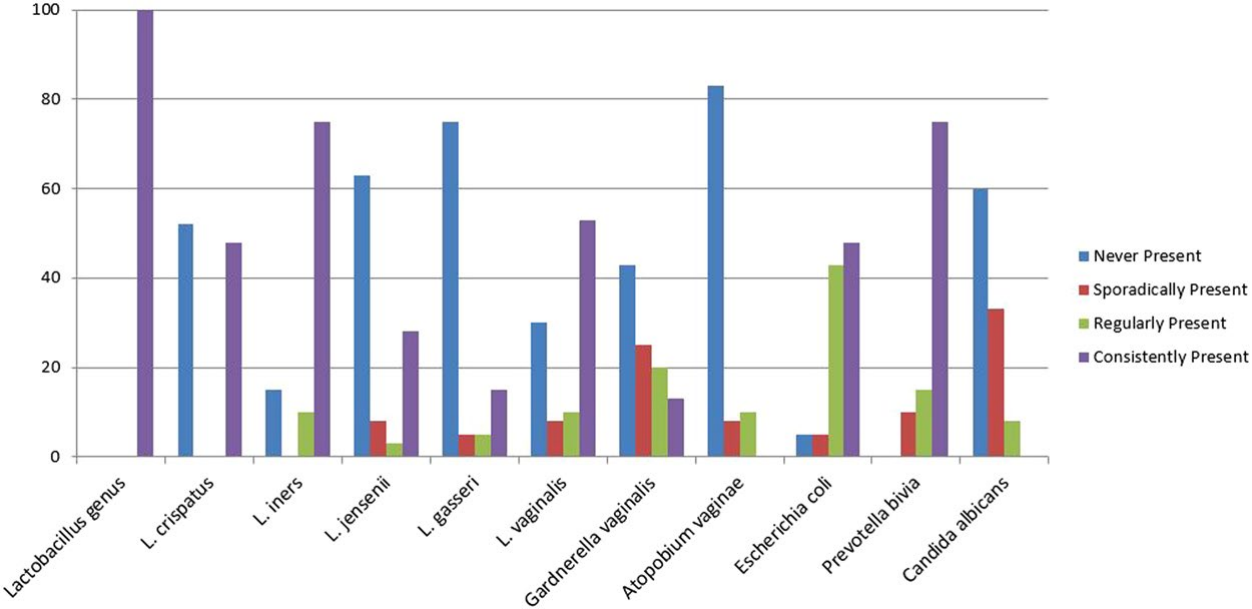
Frequency of vaginal microbiota presence over the eight week study period in women with a Nugent score of 0–3 throughout. Data in Y-axis are % of women. Sporadically present: present at 25% or fewer visits; regularly present: present at 26–74% of visits; consistently present: present at 75% or more visits.

The presence of individual VMB bacteria and *Candida albicans* was determined by qPCR and expressed as log_10_ genome equivalents (geq) per millilitre (ml). Over the five visits, presence was classified as: never present (0% of visits); sporadically present (1–25% of visits); regularly present (26–74% of visits) and consistently present (75–100% of visits). The presence of individual *Lactobacillus* species was relatively stable over the five visits in the reference group; i.e. either consistently or never present (Figs 1 and 2). This was particularly true of *L. crispatus*, which was consistently present in 47% of women or never present in 53% of women (Fig. 2). In 79% of the women with consistent *L. crispatus*, this was accompanied by a consistent or regular presence of *L. vaginalis* (Fig. 1). *L. iners* was consistently present in 75% of women and regularly present in another 10% of women. *L. iners* and *L. crispatus* did occur together at least twice in 35% of women, but women with high concentrations of *L. crispatus* had lower concentrations of *L. iners* and vice versa (Fig. 1). *C. albicans*, *L. jensenii* and *L. gasseri* were never present in 60%, 63% and 75% of the women, respectively. *Escherichia coli* was present (but always in a lower concentration than the lactobacilli) at least once in 90% of women, *Prevotella bivia* in 91% of women, *Gardnerella vaginalis* in 58% of women, and *Atopobium vaginae* in only 17% of women.

Correlates of longitudinal variations in the concentrations of VMB bacteria were assessed in mixed effects linear regression models for those VMB bacteria that were consistently present in at least 25% of the women in the reference group. Each model had one such VMB bacteria concentration as the outcome, individual women as random effects, and presence or absence of a menstrual cycle, menstrual cycle phase (follicular or luteal phase; see methods), presence of vaginal PSA, and recent vaginal cleansing as fixed effects. It is important to note that all amenorrhoeic women in this sub-study were progestin injection users. The models showed that changes in the concentrations of VMB bacteria over time were larger within women than they were between women, with the exception of *L. jensenii* (Table 2). The mean *Lactobacillus* genus concentration in amenorrhoeic women was lower (−0.55 log_10_ geq/ml; p = 0.023) than the mean concentration in women with a menstrual cycle (Table 2), with *L. crispatus* accounting for the greatest difference (Table 2). The mean *Lactobacillus* genus (−0.39 log_10_ geq/ml; p = 0.010), *L. iners* (−0.75 log_10_ geq/ml; p = 0.008) and *P. bivia* (−0.38 log_10_ geq/ml; p = 0.045) concentrations were significantly lower at visits with vaginal PSA detected (Table 2). The mean *E. coli* concentration was significantly lower at luteal phase visits compared to follicular phase visits in women with a menstrual cycle (−0.75 log_10_ geq/ml; p = 0.020). There were no significant associations between recent vaginal cleansing and concentration of any VMB bacteria (Table 2).

**Table 2:** Mean differences in VMB bacteria concentrations in women with a Nugent score of 0–3 during five visits over eight weeks by presence and phase of the menstrual cycle, presence of PSA and recent vaginal cleansing.

VMB bacteria	Women with cycle, follicular phase visits			Women with cycle, luteal phase visits vs follicular phase visits		Amenorrhoea (all visits) vs women with cycle (all visits)		Visits with PSA present vs not, among follicular phase visits with no recent vaginal cleansing reported		Visits with recent vaginal cleansing reported vs not, among follicular phase visits with PSA absent	
	Mean conc^1^	SD between^2^	SD within^2^	Mean diff^3^	p^4^	Mean diff^5^	p^4^	Mean diff^6^	p^4^	Mean diff^7^	p^4^
*Lactobacillus* genus	7.62	0.60	0.73	0.20	0.124	−0.55	**0.023**	−0.39	**0.010**	−0.27	0.146
*L. crispatus*	6.76	1.34	1.56	0.49	0.245	−1.33	0.091	−0.30	0.529	−0.05	0.939
*L. iners*	8.36	0.62	1.29	−0.25	0.356	0.08	0.811	−0.75	**0.008**	−0.32	0.272
*L. jensenii*	5.79	1.41	1.32	0.85	0.066	−0.30	0.785	0.36	0.476	0.12	0.842
*L. vaginalis*	5.84	0.92	1.31	−0.19	0.545	0.75	0.192	0.01	0.980	0.50	0.239
*E. coli*	5.26	<0.001	1.18	−0.75	**0.020**	0.14	0.637	0.10	0.757	−0.26	0.373
*P. bivia*	3.00	0.31	0.81	−0.16	0.346	0.27	0.199	−0.38	**0.045**	0.03	0.880

Abbreviations: conc = concentration; diff = difference; PSA = prostate-specific antigen; SD = standard deviation; VMB = vaginal microbiota; vs = versus.

^1^Expressed in log_10_ genome equivalents per mL (geq/ml). The expected value for women with a menstrual cycle in the follicular phase of the cycle.

^2^The between-women and within-women standard deviations.

^3^The mean difference in concentration (log_10_ geq/ml) between the luteal and follicular phases of the cycle for women with a menstrual cycle.

^4^From the mixed effects linear regression models with each item in the first column as the outcome, individual women as random effects, and fixed effects as described in the first row of the table. For women with the bacteria present during at least 75% of visits and excluding the visits during which the bacteria was absent. We only included VMB bacteria that were consistently present (in at least 75% of the visits) in at least 25% of women.

^5^The mean difference in concentration (log_10_ geq/ml) between women with amenorrhoea (all visits) and women with a cycle (all visits).

^6^The mean difference in concentration (log_10_ geq/ml) between visits with PSA present versus not present for visits with the same presence and phase of menstrual cycle and the same vaginal cleansing status.

^7^The mean difference in concentration (log_10_ geq/ml) between visits at which reporting recent vaginal cleansing was reported versus not reported among visits with the same presence and phase of menstrual cycle and the same PSA status.

### Vaginal immune mediators over time (reference group)

Concentrations of various cytokines, chemokines, and growth factors were measured in cervicovaginal lavages (CVLs) and expressed in log_10_ pg/ml (see methods). Mixed effects linear regression models with each immune mediator concentration as the outcome, individual women as random effects, and menstrual cycle presence and phase as fixed effects showed that changes in concentrations of immune mediators over time were larger within women than they were between women, with the exception of interleukin (IL)−1α (Table 3). The mean IL-1α concentration was significantly higher in luteal phase relative to follicular phase visits (0.16 log_10_ pg/ml; p = 0.004) but mean IL-6 (−0.26 log_10_ pg/ml; p < 0.001), CC chemokine macrophage inflammatory protein (MIP)−1β (−0.26 log_10_ pg/ml; p < 0.001) and granulocyte colony-stimulating factor (G-CSF) concentrations (−0.26 log_10_ pg/ml; p = 0.007) were significantly lower. Mean concentrations of IL-8 (0.28 log_10_ pg/ml; p = 0.016), IL-12(p70) (0.15 log_10_ pg/ml; p = 0.038) and MIP-1β (0.34 log_10_ pg/ml; p = 0.013) were higher in amenorrhoeic women compared to women with a menstrual cycle. Further mixed effects linear regression models with each immune mediator as the outcome, individual women as random effects, and presence and phase of the menstrual cycle as fixed effects, were fitted with the following additional fixed effects added (in separate models): vaginal pH category (<4.0, 4.0–4.5, >4.5); presence of abnormal vaginal discharge, cervical mucus, a cervical epithelial finding, or vaginal PSA; and recent vaginal cleansing. Visits with PSA detected had significantly higher mean concentrations of IL-6, IL-12(p70), and CXC chemokines interferon (IFN)-γ-inducible protein (IP-10); visits with a higher vaginal pH had a higher mean concentration of IL-1RA and a lower mean concentration of secretory leucocyte peptidase inhibitor (SLPI); visits with abnormal vaginal discharge had lower mean concentrations of IL-1α, IL-1RA, GM-CSF and elafin; visits with cervical mucus had a lower mean concentration of elafin; and visits with cervical epithelial findings had a lower mean concentration of granulocyte macrophage colony stimulating factor (GM-CSF) (Table 3). Recent vaginal cleansing was not significantly associated with concentrations of any of the immune mediators (data not shown).

**Table 3:** Mean differences in immune mediator concentrations in women with a Nugent score of 0–3 during five visits over eight weeks by presence and phase of the menstrual cycle (A), clinical characteristics and presence of PSA (B).

**A. Immune mediators**	**Women with cycle**, **follicular phase visits**			**Women with cycle**, **luteal phase** ** vs follicular phase visits**		**Amenorrhoea** ** (all visits) vs women with cycle (all visits)**					
	Mean conc^1^	SD between^2^	SD within^2^	Mean diff^5^	p^3^	Mean diff^6^	p^3^				
Total protein	8.17	0.21	0.24	0.03	0.447	0.15	0.071				
IL-1α	1.10	0.36	0.30	0.16	**0.004**	0.15	0.253				
IL-1β	0.66	0.39	0.51	−0.06	0.521	0.27	0.081				
IL-6	0.78	0.42	0.50	−0.32	**<0.001**	0.31	0.055				
IL-8	2.01	0.30	0.34	−0.06	0.334	0.28	**0.016**				
IL-12(p70)	−0.05	0.16	0.30	−0.00	0.949	0.15	**0.038**				
IL-1RA	4.73	0.48	0.57	0.12	0.257	0.07	0.708				
IP-10	2.52	0.29	0.42	−0.09	0.258	0.17	0.155				
MIP-1β	0.81	0.36	0.37	−0.26	**<0.001**	0.34	**0.013**				
GM-CSF	0.32	0.12	0.35	0.02	0.741	0.06	0.340				
G-CSF	1.81	0.48	0.53	−0.26	**0.007**	0.33	0.071				
Elafin	5.07	0.34	0.37	0.01	0.824	−0.12	0.333				
SLPI	4.75	0.33	0.41	0.04	0.611	0.05	0.666				
**B**. **Immune mediators**	**Visits with** **vaginal pH** **4.0–4.5 or >4.5 vs <4**			**Visits with** **vaginal discharge** **vs not**		**Visits with** **cervical mucus** **vs not**		**Visits with** **cervical epith findings** **vs not**		**Visits with** **PSA present** **vs not**	
	**4.0–4.5 Mean diff** ^**8**^	**>4.5 Mean diff** ^**8**^	**p** ^**4**^	**Mean diff** ^**9**^	**P** ^**4**^	**Mean diff** ^**10**^	**p** ^**4**^	**Mean diff** ^**11**^	**p** ^**4**^	**Mean diff** ^7^	**p** ^**4**^
Total protein	−0.00	−0.16	**0.014**	−0.07	0.150	−0.04	0.386	0.08	0.235	0.02	0.650
IL-1α	0.09	0.01	0.187	−0.13	**0.024**	−0.09	0.080	−0.09	0.266	0.10	0.095
IL-1β	0.14	0.07	0.366	0.07	0.464	−0.04	0.601	−0.07	0.638	0.09	0.390
IL-6	0.08	0.22	0.170	0.16	0.098	0.04	0.671	0.00	0.988	0.21	**0.032**
IL-8	0.09	0.06	0.409	0.06	0.355	−0.02	0.684	−0.08	0.395	0.09	0.188
IL-12(p70)	0.11	0.14	0.097	0.06	0.280	0.01	0.876	0.11	0.154	0.19	**0.001**
IL-1RA	0.05	0.35	**0.015**	−0.25	**0.025**	−0.14	0.132	0.05	0.734	−0.02	0.840
IP-10	0.06	−0.13	0.064	−0.04	0.611	−0.08	0.981	0.00	0.981	0.20	**0.014**
MIP-1β	0.14	0.14	0.163	−0.00	0.989	0.03	0.601	−0.08	0.434	0.13	0.085
GM-CSF	0.01	−0.09	0.304	−0.19	**0.001**	−0.01	0.926	−0.18	**0.040**	−0.07	0.251
G-CSF	0.06	0.03	0.844	−0.01	0.942	0.11	0.215	−0.07	0.626	0.17	0.117
Elafin	0.04	−0.02	0.678	−0.23	**0.001**	−0.13	**0.033**	0.10	0.349	0.02	0.773
SLPI	0.01	−0.20	**0.034**	0.01	0.899	0.07	0.288	0.08	0.489	0.14	0.103

Abbreviations: clin char = clinical characteristics; conc = concentration; diff = difference; epith = epithelial; G-CSF = granulocyte colony stimulating factor; GM-CSF = granulocyte macrophage colony stimulating factor; IL = interleukin; IP-10 = interferon-inducible protein 10; MIP-1β = macrophage inflammatory protein 1β; PSA = prostate-specific antigen; SD = standard deviation; SLPI = secretory leukocyte protease inhibitor; VMB = vaginal microbiota; vs = versus.

^1^Expressed in log_10_ pg/ml. The expected value for women with a menstrual cycle in the follicular phase of the cycle.

^2^The between-women and within-women standard deviations for women in the model with presence and phase of the cycle as fixed effects.

^3^From mixed effects linear regression models with each item in the first column as the outcome, individual women as random effects, and including presence and phase of the menstrual cycle as fixed effects.

^4^From mixed effects linear regression models (separate model for each clinical characteristic and PSA) with each item in the first column as the outcome, individual women as random effects, and including the item in the first row as fixed effects, and controlled for presence and phase of the menstrual cycle. A model with recent vaginal cleansing (the evening or morning before the visit) as fixed effect controlled for presence and phase of the menstrual cycle was also fitted but the data are not shown because none of the findings were statistically significant. The clinical characteristics are clinician-observed during speculum examination.

^5^The mean difference in concentration (log_10_ pg/ml) between luteal and follicular phase visits in women with a menstrual cycle.

^6^The mean difference in concentration (log_10_ pg/ml) between women with amenorrhoea (all visits) and women with a cycle (all visits).

^7^The mean difference in concentration (log_10_ pg/ml) between visits with PSA present versus absent, for visits with the same presence and phase of menstrual cycle.

^8^The mean difference in concentration (log_10_ pg/ml) between visits at which the vaginal pH was 4.0–4.5, or >4.5, each compared to <4, for visits with the same presence and phase of menstrual cycle.

^9^The mean difference in concentration (log_10_ pg/ml) between visits with vaginal discharge present versus absent, for visits with the same presence and phase of menstrual cycle.

^10^The mean difference in concentration (log_10_ pg/ml) between visits with cervical mucus present versus absent for visits with the same presence and phase of menstrual cycle.

^11^The mean difference in concentration (log_10_ pg/ml) between visits with cervical epithelial findings present versus absent, for visits with the same presence and phase of menstrual cycle. Cervical epithelial findings included abrasions, oedema, ecchymosis, petechiae, erythema, and ulcers.

### VMB bacteria, Candida, and immune mediators over time (incident BV group)

**Figure 3:**
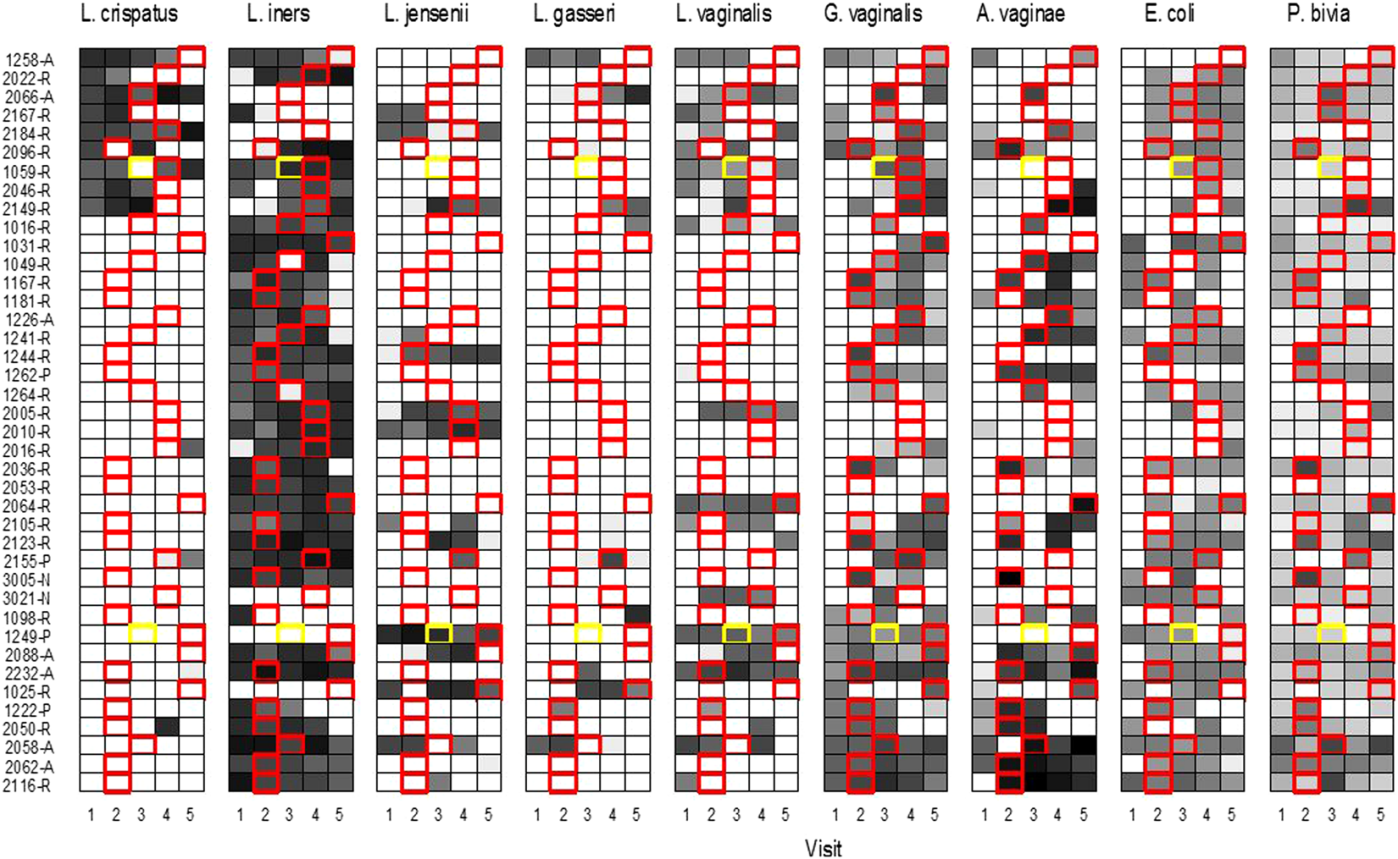
Presence/absence and concentration of vaginal microbiota over the eight week study period in women with incident BV (Nugent 7–10). Each box depicts one visit for a particular woman. The shading of the box indicates the concentration level (in log_10_ geq/ml) of each taxon with darker colours depicting a higher concentration. If the species was absent the box is white. Boxes bordered by a red line are the first BV visit for that woman. Boxes in yellow denote visits with an intermediate Nugent score of 4–6 if present before development of BV.

All women in this cohort had a Nugent score of 0–3 at visit 1 (enrolment). The first visit during which BV was diagnosed was visit 2 in 16 women, visit 3 in seven women, visit 4 in 11 women, and visit 5 in six women (Fig. 3). Two women had an intermediate VMB (Nugent score 4–6) prior to an incident BV visit. At the first visits during which BV was detected, the mean concentrations of *Lactobacillus* genus (−1.51 log_10_ geq/ml; p = 0.005) and *L. vaginalis* (−1.35 log_10_ geq/ml; p = 0.021) were statistically significantly lower, and the mean concentrations of *G. vaginalis* (2.84 log_10_ geq/ml; p < 0.001), *A. vaginae* (3.92 log_10_ geq/ml; p < 0.001), and *P. bivia* (1.38 log_10_ geq/ml; p = 0.003) higher, than the mean concentrations at the preceding visit. (Table 4). *C. albicans*, *L. jensenii* and *L. gasseri* were never present at any visits in 58%, 60% and 73% of the women, respectively. Mean concentrations of IL-1β (0.66 log_10_ pg/ml; p = 0.003) and IL-12(p70) (0.22 log_10_ pg/ml; p = 0.024) were significantly increased, and mean concentrations of IP-10 (−0.39 log_10_ pg/ml; p = 0.046), elafin (−0.26 log_10_ pg/ml; p = 0.010), and total protein (−0.17 log_10_ pg/ml; p = 0.026) significantly decreased, at the first BV incident visit (Table 4).

**Table 4:** Differences in VMB bacteria and immune mediator concentrations in 40 women with incident BV between the visit before the incident BV visit and the incident BV visit.

	Visit before the first incident BV visit	First incident BV visit^1^	Mean concentration difference	P^3^
	Mean concentration^2^	Mean concentration^2^		
**VMB bacteria**				
Triple taxa qPCR vaginal health score^4^	5.44	0.20	−5.24	**<0.001**
*Lactobacillus* genus	7.60	6.09	−1.51	**0.005**
*L. crispatus*	1.91	0.86	−1.05	0.250
*L. iners*	5.84	4.85	−0.99	0.080
*L. vaginalis*	2.76	1.42	−1.35	**0.021**
*G. vaginalis*	2.11	4.95	2.84	**<0.001**
*A. vaginae*	0.20	4.12	3.92	**<0.001**
*E. coli*	3.04	2.75	−0.29	0.709
*P. bivia*	1.76	3.13	1.38	**0.003**
**Immune mediators**				
Total protein	8.29	8.12	−0.17	**0.026**
IL-1α	1.23	1.49	0.26	0.157
IL-1β	0.69	1.35	0.66	**0.003**
IL-6	0.77	1.06	0.28	0.081
IL-8	2.07	2.29	0.22	0.104
IL-12(p70)	−0.00	0.22	0.22	**0.024**
IL-1RA	4.71	4.99	0.28	0.250
IP-10	2.63	2.25	−0.39	**0.046**
MIP-1β	0.70	0.89	0.18	0.372
GM-CSF	0.34	0.28	−0.06	0.157
G-CSF	1.96	1.96	0.11	0.579
Elafin	5.07	4.81	−0.26	**0.010**
SLPI	4.80	4.62	−0.18	0.287

Abbreviations: BV = bacterial vaginosis; G-CSF = granulocyte colony stimulating factor; GM-CSF = granulocyte macrophage colony stimulating factor; IL = interleukin; IP-10 = interferon-inducible protein 10; MIP-1β = macrophage inflammatory protein 1β; SLPI = secretory leukocyte protease inhibitor; VMB = vaginal microbiota.

^1^The first incident BV visit was visit 2 for 16 women, visit 3 for 7 women, visit 4 for 11 women and visit 5 for 6 women.

^2^Expressed in log_10_ genome equivalents per mL (geq/ml) for VMB bacteria and log_10_ pg/ml for immune mediators.

^3^Wilcoxon signed rank tests. ^4^log_10_ geq/ml (*Lactobacillus* genus)−log_10_ geq/ml (*G. vaginalis* + *A. vaginae*).

### VMB bacteria and immune mediator associations over time (both groups)

In mixed effects linear regression models including all 80 women and controlled for presence and phase of menstrual cycle and PSA presence, a higher ‘composite qPCR vaginal health score’ was associated with a higher IP-10 concentration (2.53 log_10_ pg/ml; p < 0.001) and lower IL-1α (−1.14 log_10_ pg/ml; p = 0.005), IL-8 (−1.55 log_10_ pg/ml; p = 0.002), and IL-12(p70) (−1.80 log_10_ pg/ml; p < 0.001) concentrations (Table 5). This vaginal health score was calculated as [log_10_ geq/ml (*Lactobacillus* genus)−log_10_ geq/ml (*G. vaginalis* + *A. vaginae*)] and a higher score therefore suggests better vaginal health^26^. The *Lactobacillus* genus concentration (which is one component of the vaginal health score) showed a similar pattern except that it was not significantly associated with the IL-1α concentration and the reduction in IL-12(p70) concentration did not reach statistical significance. The *L. crispatus* and *L. vaginalis* concentrations were not significantly associated with any immune mediator concentrations over time. The *L. iners* concentration was significantly positively associated with IP-10 and IL-8 concentrations and negatively associated with IL-1α concentration. The *P. bivia* concentration was positively associated with the IL-1α and IL-8 concentrations and negatively associated with the IP-10 concentration, and the *E. coli* concentration was positively associated with the IL-8 concentration.

**Table 5:** Longitudinal associations between VMB bacteria and immune mediator concentrations among all visits of all 80 women (with Nugent 0–3 throughout and with incident BV) over the eight week study period.

	IL-1α		IL-8		IL-12(p70)		IP-10		Elafin		PSA		Amenorrhoea		Luteal phase	
	Est.^1^	p^1^	Est.^1^	p^1^	Est.^1^	p^1^	Est.^1^	p^1^	Est.^1^	p^1^	Est.^1^	p^1^	Est.^1^	p^1^	Est.^1^	p^1^
Triple taxa qPCR vaginal health score^2^	−1.14	**0.005**	−1.55	**0.002**	−1.80	**<0.001**	2.53	**<0.001**	0.34	0.383	−1.07	**0.002**	0.80	0.160	0.52	0.118
*Lactobacillus* genus^3^	0.02	0.939	−0.75	**0.003**	−0.42	0.105	0.92	**<0.001**	−0.21	0.301	−0.49	**0.005**	−0.37	0.135	0.34	0.054
*L. crispatus* ^3^	0.49	0.353	−0.49	0.466	−0.05	0.921	0.79	0.098	−0.39	0.317	−0.55	0.218	−1.36	0.053	0.29	0.452
*L. iners* ^3^	−0.58	**0.020**	0.77	**0.010**	−0.50	0.108	0.49	**0.011**	−0.42	0.078	−0.38	0.076	−0.26	0.280	−0.10	0.696
*L. vaginalis* ^3^	0.24	0.563	0.60	0.289	−0.25	0.562	−0.43	0.276	−0.00	0.991	−0.14	0.688	0.18	0.722	−0.06	0.849
*P. bivia* ^3^	0.52	**0.004**	0.78	**0.001**	0.03	0.917	−0.69	**<0.001**	0.20	0.296	−0.05	0.759	−0.29	0.214	−0.19	0.268
*E. coli* ^3^	−0.00	0.995	0.55	**0.049**	−0.60	0.054	0.28	0.139	−0.05	0.832	0.29	0.160	−0.05	0.812	−0.14	0.491

Abbreviations: BV = bacterial vaginosis; Est = model estimate; IL = interleukin; IP-10 = interferon-inducible protein 10; qPCR = quantitative polymerase chain reaction; PSA = prostate-specific antigen; VMB = vaginal microbiota; vs = versus.

^1^From mixed effects multiple regression models with each item in the first column as the outcome, individual women as random effects, and fixed effects for all variables in the first row.

^2^log_10_ geq/ml (*Lactobacillus* genus)−log_10_ geq/ml (*G. vaginalis* + *A. vaginae*).

^3^For women with the bacteria present during at least 75% of visits and excluding the visits during which the bacteria was absent. We only included VMB bacteria that were consistently present (in at least 75% of the visits) in at least 25% of women.

## Discussion

In this longitudinal study of young sub-Saharan African women, we confirmed that a Nugent score of 0–3 over an eight week period was associated with consistently high concentrations of *Lactobacillus* species (regularly accompanied by much lower concentrations of the BV-associated bacteria *G. vaginalis*, *A. vaginae*, and *P. bivia* and the pathobiont *E. coli*), whereas incident BV was associated with significantly reduced concentrations of lactobacilli and increased concentrations of *G. vaginalis*, *A. vaginae* and *P. bivia*, but not *E. coli*. In women with a normal VMB throughout the study, VMB variations were larger within women over time than between women, as has been seen in other studies^19^. *L. iners* and *L. crispatus* were the dominant lactobacilli in our study, as has been seen in studies enrolling Caucasian European and American women^2,27,28^. However, the following of our findings have not been reported in those studies: *L. crispatus* was often accompanied by *L. vaginalis*; *L. jensenii* and *L. gasseri* were never present in most women; and *E. coli* was regularly present in almost all women. Another important pathobiont in the vaginal niche is *Streptococcus agalactiae*, and unfortunately, we only have qPCR data for that organism at baseline^29^. In a cross-sectional baseline analysis of all 430 women in the Vaginal Biomarkers Study using qPCRs, 16% had *S. agalactiae* ^29^ and 28% *E. coli* in their VMB^16^. The limited number of other molecular VMB studies that reported on *S. agalactiae* and *E. coli* carriage showed varying results, with generally lower detection in studies that employed 16 S sequencing compared to qPCR^18,30,31^. Vaginal carriage of these pathobionts should be further investigated, preferably by qPCR in longitudinal studies, given their associations with vaginitis, reproductive health, and neonatal meningitis and sepsis^32^.

In women with a normal VMB throughout the study, variations in concentrations of soluble immune mediators were greater within women over time than between women, which is in agreement with other studies^12,33,34^. In the women developing BV, incident BV was associated with increased concentrations of proinflammatory cytokines and decreased concentrations of the antiprotease elafin and IP-10. Other studies have reported similar proinflammatory profiles associated with BV, as well as increased proteolytic activity^35–38^. IP-10 findings across studies are more difficult to interpret (see below). It should be noted that incident or recurrent urogenital infections other than BV could have been responsible for some of the variation in immune mediators seen. However, none of the 80 women in this study had symptomatic vaginal candidiasis throughout the study and *C. albicans*, detected only occasionally and never more than twice in the same women, was present in low concentrations in the majority of women. Furthermore, we screened all women for STIs at baseline and selected women without STIs (with the exception of chronic HSV-2 infection) for this sub-study. Only women with clinician-observed signs of urogenital infections during the eight-week follow-up period were retested for STIs, but such clinician-observed signs were rare (Table 1). We therefore believe that incident or recurrent STIs during the eight-week follow-up period were uncommon.

We assessed several other potential correlates of VMB bacteria as well as immune mediator concentration variations in women with a normal VMB throughout the study: the presence and phase of the menstrual cycle, the presence of PSA as a marker of recent sex, and recent vaginal cleansing. Amenorrhoeic women had a reduced concentration of lactobacilli, (notably *L. crispatus*), compared to women with a menstrual cycle even after controlling for PSA presence and recent vaginal cleansing, and this may be due to the induction of a hypo-oestrogenic state during injectable progestin use. Current evidence suggests that the VMB destabilising effect of hypo-oestrogenism in these women is larger than any potential protective effect associated with the absence of regular menstrual bleeding^30^. Amenorrhoeic women also had increased concentrations of several proinflammatory immune mediators. This is in agreement with results from two African studies (Tanzania and South Africa/Kenya)^39,40^ but in contrast to the results of a recent study in Kenyan women that showed sustained decreases in IL-6, IL-8, and IL-1RA after initiation of depot medroxyprogesterone acetate (DMPA) injectable contraception^41^. The comparison groups in these studies differed, with our study comparing amenorrhoeic injectable progestin users with all other women, the Tanzanian and South Africa/Kenya studies comparing current DMPA users with women not using hormonal contraception, and the Kenyan study comparing women before and after initiation of DMPA use. It is possible that DMPA use is immunosuppressive initially as it binds to the corticosteroid receptor with an affinity similar to that of cortisol^42^, but becomes proinflammatory with prolonged use due to increasing hypo-oestrogenism which in turn can lead to VMB dysbiosis and vaginal wall atrophy.

We did find some differences in VMB bacteria and the concentration of immune mediators in samples collected during luteal phase visits compared to follicular phase visits, but these patterns were not consistent. While levels of both oestrogen and progesterone are higher in the luteal phase than the follicular phase of the menstrual cycle, we sampled women around days 9 and 23 of their cycles, and these time points do not correspond with peak hormone levels. Oestrogen in particular is known to associate with higher concentrations of lactobacilli^17,43,44^. We did see increases in concentrations of *Lactobacillus* genus, *L. crispatus*, and *L. jensenii* at luteal phase visits, but these did not reach statistical significance. The differences in mean immune mediator concentrations between the luteal and follicular phases that we observed were not seen in the earlier mentioned Tanzanian study, and that study assessed menstrual cycle stage more carefully by urine pregnanediol 3-glucuronide testing^39^.

Vaginal sex in the last 24–48 hours as measured by the presence of PSA in vaginal swab eluates was associated with concentrations of various lactobacilli, with *L. iners* showing the greatest reduction. PSA presence was also associated with higher concentrations of IL-6, IL-12(p70), and IP-10. Similar effects of recent vaginal sex on the VMB have been previously reported by us^33^ and by others^17,45–47^. A direct effect was demonstrated *in vitro* when seminal plasma was co-cultured with cervical epithelial cells^48^. The VMB-destabilising and proinflammatory effects of sexual activity are likely due to the direct effect of seminal fluid as condom use seems to prevent them^45,49^. Recent vaginal cleansing was not significantly associated with any changes in VMB bacteria or immune mediator concentrations in any of our analyses.

Using data from all 80 women, we investigated the direct associations between the concentrations of VMB bacteria and vaginal immune mediator concentrations over time while controlling for presence and phase of the menstrual cycle and PSA presence. Perhaps the most significant finding was that a higher ‘composite qPCR vaginal health score’ (suggesting better vaginal health) was associated with decreased concentrations of all three modelled proinflammatory cytokines (IL-1α, IL-8, and IL-12(p70)) and an increased concentration of IP-10. Unfortunately, the sample size of this sub-study was small; some statistically significant associations in the cross-sectional analyses of the Vaginal Biomarkers Study baseline data showed the same trends as this sub-study but did not reach statistical significance^22^. When interpreting the cross-sectional^16,22^ and longitudinal data together, we conclude that *Lactobacillus* species are associated with an increase in IP-10 and reductions (*L. crispatus*, *L. vaginalis*) or no change in multiple proinflammatory cytokines; BV-associated bacteria are associated with a decrease in IP-10 and increases in multiple proinflammatory cytokines; and *E. coli* and *S. agalactiae* are associated with increases in IP-10 and multiple proinflammatory cytokines (the *S. agalactiae* data are unpublished). A cross-sectional Canadian study employing 16 S sequencing to characterise the VMB reported very similar results: a decrease in IP-10 and increases in multiple proinflammatory cytokines in women with BV (community state type (CST)-4), and no inflammation but an increase in IP-10 in women with a *L. iners* dominated VMB (CST-3)^50^. A longitudinal South African study also found significant increases in multiple proinflammatory cytokines at visits during which vaginal dysbiosis was detected, but no association with IP-10^12^. IP-10 (also known as CXCL10) is induced by type I and II interferons and TNF-α and is a ligand for the CXCR3 receptor^51–53^. IP-10 levels are generally elevated in uncontrolled viral infection, but a reduction of IP-10 levels by pathogenic bacteria, and particularly combinations of bacteria, has been described before^54–57^. The significance of this remains unclear. A recent study among women in South Africa by Masson *et al*. found that increased IL-1β and reduced IP-10 concentrations in female genital secretions of HIV-negative women predicted the presence of BV and/or other treatable discharge-causing STIs^37^. The combination of these two biomarkers identified a significantly higher proportion (77%) of women with BV and treatable STIs than clinical criteria (19%). Consequently, the authors suggested to explore the use of those biomarkers in the detection of BV and discharge causing STIs^38^.

Our study had some limitations. Unfortunately, we could not afford to quantify all relevant bacteria and immune mediators in all longitudinal samples from all participants of the Vaginal Biomarkers Study. The current sub-study design was considered a next best but feasible alternative. This design required us to select women based on longitudinal Nugent scores, which are a cruder way of classifying VMBs than the molecular methods we employed in the sub-study. However, multiple studies have shown a good correlation between the two methods in classifying woman as having a lactobacilli-dominated or dysbiotic VMB^1^, with the molecular testing adding nuance. Our sub-study design also reduced our statistical power, especially related to the potential effects of VMB minority species. Due to the stringent selection criteria, our results may not be generalisable to all women.

In conclusion, our well-controlled longitudinal data confirm the inflammatory nature of anaerobic vaginal dysbiosis and *E. coli* colonisation, recent vaginal sex, and progestin-injectable use. While anaerobic vaginal dysbiosis or BV is by far the most common vaginal dysbiosis, high abundance of *E. coli*, *S. agalactiae*, and other pathobionts as a distinct inflammatory vaginal dysbiosis deserves further study. The roles of a selection of the vaginal mediators (IL-1α, IL-1β, IL-8, IL-12, IP-10) with or without the composite qPCR vaginal health score as predictive biomarkers for the above conditions warrant further investigation.

## Methods

### Ethical approvals

The study protocol was approved by the Ethical Review Committee, Kenyatta National Hospital, Kenya; the Human Research Ethics Committee (Medical), University of the Witwatersrand, South Africa; the Rwanda National Ethics Committee, Rwanda; the Institutional Review Board of the Institute of Tropical Medicine (ITM), Belgium; and the ethics committees of the Ghent University Hospital in Ghent and the Antwerp University Hospital in Antwerp, Belgium. In addition the study was approved by the National Council of Science and Technology, Kenya; and the National Health Research Committee, Rwanda. All methods described below were performed in accordance with the relevant guidelines and regulations.

### Study participants and clinic visits

This paper describes a longitudinal analysis of 80 women who were part of the Vaginal Biomarkers Study, which enrolled a total of 430 women at three study sites: the International Centre of Reproductive Health Kenya in Mombasa, Kenya; the Wits Reproductive Health and HIV Institute in Johannesburg, South Africa and Rinda Ubuzima in Kigali, Rwanda. In the original study design, women were recruited into predefined groups: HIV-negative adult women (N = 219), adolescents (N = 60), and pregnant women (N = 60) in Kenya and South Africa; HIV-positive women (N = 30) and HIV-negative sex workers (N = 30) in Rwanda; and women engaging in traditional vaginal practices in South Africa (N = 31). For the current analysis, we selected 40 women who had a normal VMB (defined as a Nugent score of 0–3) at five consecutive visits over eight weeks and 40 women who had a normal VMB at baseline and incident BV (defined as a Nugent score of 7–10) at one of the four follow-up visits over eight weeks. Additional selection criteria were: none of the relevant samples were missing; women tested negative for pregnancy, HIV, syphilis, *N. gonorrhoeae*, *C. trachomatis*, *T. vaginalis*), and vaginal candidiasis at baseline and did not become HIV-positive or pregnant (reference group) during follow-up; and reported not to engage in traditional vaginal practices (such as the use of cloth, lemon juice or detergents inside the vagina) at baseline. Women with positive HSV-2 serology at baseline were included due to the high prevalence of 34%. A total of 54 and 48 women qualified for the reference and incident BV groups, respectively, and 40 women were selected for each group from among the qualifying women at random. No matching was done.

Women were followed for five consecutive visits over eight weeks. The visits were tightly scheduled around the menstrual cycle with the enrolment visit (visit 1) scheduled shortly after the last day of the menstrual period on day 9 (±2 days) of the cycle; the absence of menses was verified during vaginal examination at this visit. The next four visits were scheduled with two week intervals over two menstrual cycles (visits 2–5). Thus, visits 3 and 5 coincided with day 9 (±2 days) of the menstrual cycle (the follicular phase) and visits 2 and 4 with day 23 (±2 days) or the luteal phase. The same visit schedule was followed for women using hormonal contraception, including those who were amenorrhoeic due to progestin-injectable use. At baseline, eligible women interested in participating provided written informed consent, were interviewed about sociodemographic and behavioural characteristics, underwent a physical and vaginal examination, and were tested for HIV and the above-mentioned reproductive tract infections. Interviews and vaginal examinations were also done at all subsequent visits.

### Sample collection

At each of the five visits included in this sub-study, the following samples were collected before any other procedures in the following order: two sterile flocked swabs (Copan Diagnostics, Inc., Murrieta, CA) that were rotated against the mid-portion of the vaginal wall under visual inspection, dipped in the posterior fornix and carefully removed to prevent contamination; and a CVL that was obtained by gently flushing 10 ml normal saline through the speculum and aspirating the fluid from the posterior fornix. At each study site, one trained clinician performed all the examinations using one standard operating procedure to minimise inter-clinician variability.

### Sample processing

CVLs were collected in 15 ml falcon tubes, kept on ice for transport (2–8 °C), and processed within a maximum of one hour after collection. CVLs were centrifuged at 1,000 x g for 10 minutes at 4 °C and supernatants (~9 ml) were aliquoted into three fractions; two of approximately 4 ml each and one of 1 ml. The aliquots were stored at −80 °C locally. CVLs and vaginal swabs, frozen at −80 °C, were shipped in batches using a temperature-monitored dry shipper to the central laboratory at the ITM in Antwerp, Belgium, where they were stored at −80 °C before analysis of soluble immune mediators and VMB bacteria.

### Characterisation of vaginal microbiota

Vaginal Gram-stained slides were examined and scored at the ITM using the Nugent method^14^. qPCR was performed on extracted DNA from vaginal swab eluates for the following ten species and one genus: *Lactobacillus* genus, *L. crispatus*, *L. gasseri*, *L. iners*, *L. jensenii*, *L. vaginalis*, *A. vaginae*, *G. vaginalis*, *E. coli*, *P. bivia*, and *C. albicans* and in duplicate at the ITM and at the University of Ghent, Belgium, as previously described^16,18^. The number of organisms was expressed as genome equivalents per ml (geq/ml); the genomic concentration was calculated using the described genomic sizes of the type strains.

### Quantification of soluble immune mediators in CVLs

Concentrations of the cytokines IL-1α, IL-1β, IL-6 and IL-12(p70), MIP-1β, IP-10 and IL-8, and growth factors GM-CSF and G-CSF in CVLs were measured at the ITM using the Bio-Plex™ human cytokine assay kit (Bio-Rad Laboratories NV-SA, Nazareth, Belgium) as previously described^33^. Elafin, SLPI, IL-1RA and the total protein concentration in CVLs were measured in the Laboratory of Genital Tract Biology, Brigham and Women’s Hospital, Boston, MA, USA. Elafin and SLPI were quantified using ELISA kits from R&D Systems (Minneapolis, MN) following manufacturers’ instructions. IL-1RA was measured using the Meso Scale Discovery (MSD) multiplex platform and Sector Imager 2400 (MSD, Gaithersburg, MD). The MSD Discovery Workbench Software was used to convert relative luminescent units into protein concentrations (pg/ml) using interpolation from several log calibrator curves. Total protein in CVLs was determined by a bicinchoninic acid (BCA) assay (Thermo Scientific, Rockford, IL) using the Victor 2 counter. Optical densities were read at 450 nm with a second reference filter of 570 nm using a Victor2 multi-label reader and WorkOut Software (PerkinElmer, Waltham, MA).

### Prostate-specific antigen detection

PSA was measured in vaginal swab eluates using the Seratec^®^ PSA semiquant assay (Seratec Diagnostica, Göttingen, Germany). A volume of 150 µl of the eluate, in diluted phosphate buffered saline (1,200 μl; 1 part phosphate buffered saline and 9 parts saline, pH 7.4), was centrifuged for 10 min at 13,000 x g. After centrifugation, 120 µl of supernatant was used for testing according to the manufacturer’s instructions.

### Data analysis

Statistical analyses were performed using Stata 13 (StataCorp, College Station, TX), SAS 9.4 (SAS Institute Inc, Cary, NC) and R 3.0.1 (The R Foundation, Vienna, Austria). Over the five study visits, the detection of individual VMB bacteria by qPCR was classified as follows: never present; sporadically present (present at 25% or fewer visits); regularly present (present at 26–74% of visits) and consistently present (present at 75% or more visits). The concentrations of VMB bacteria (in geq/ml) and immune mediators (in pg/ml) were log_10_ transformed in all analyses.

For the women with a normal VMB throughout the study: Longitudinal variations in the concentrations of VMB bacteria were assessed in mixed effects linear regression models for those VMB bacteria consistently present in at least 25% of the women with a normal VMB throughout the study. All models included one VMB bacteria as the outcome and individual women as random effects. We added the following fixed effects: sampling in the luteal (visits 2 and 4) versus follicular phase (visits 1, 3 and 5) of the menstrual cycle, the absence (amenorrhoea due to progestin-injectable use) or presence of a menstrual cycle (either a natural cycle or regular withdrawal bleeds during combined contraceptive use), presence of PSA as a marker of sex within the last 24–48 hours^23,24^, and recent vaginal cleansing (the evening or morning just prior to the study visit).

For the women with a consistently normal VMB, mixed effects linear regression models were also fitted with each immune marker concentration as the outcome, individual women as random effects, and including presence and phase of the menstrual cycle as fixed effects. Further mixed effects linear regression models for each marker as outcome, controlled for presence and phase of the menstrual cycle, were fitted separately for the following covariates: vaginal pH category (<4.0, 4.0–4.5, >4.5), presence of clinician-observed abnormal vaginal discharge, cervical mucus, a cervical epithelial finding (abrasion, laceration, ecchymosis, petechiae, erythema, or ulcer), vaginal cleansing, and PSA. These covariates were selected based on previous cross-sectional analyses in the same study population^16,21,22^ and based on the published literature^7,39^.

In the women with incident BV, we assessed the mean change in concentrations of VMB bacteria and immune mediators between the visit preceding the first incident BV visit and the first incident BV visit using Wilcoxon signed rank tests. Furthermore, the direct associations between VMB bacteria concentrations (for VMB bacteria consistently present in at least 25% of the women) and immune marker concentrations (IL-1α, IL-8, IL-12, IP-10, and elafin) were determined in mixed effects linear regression models in all 80 women. All models included the concentration of an individual VMB bacterium as the outcome, individual women as random effects, and the following fixed effects: each immune mediator concentration, PSA presence, and presence and phase of menstrual cycle. We also considered a ‘triple taxa qPCR vaginal health score’ based on the concentrations of three key VMB bacteria [log_10_ (*Lactobacillus* genus)−log_10_ (*G. vaginalis* + *A. vaginae*)] as the outcome because this score was shown to be the best indicator of vaginal health in the Vaginal Biomarkers Study^26^.

### Data availability

According to the Institute of Tropical Medicine’s policy, all data are available from the Institute of Tropical Medicine Institutional Data Access for researchers who meet the criteria for access to confidential data. Requests for data access can be made by emailing Mr. Jef Verellen, Quality Specialist at ITMresearchdataaccess@itg.be.
